# Rainy season onset mainly drives the spatiotemporal variability of spring vegetation green-up across alpine dry ecosystems on the Tibetan Plateau

**DOI:** 10.1038/s41598-020-75991-w

**Published:** 2020-11-02

**Authors:** Xiang Li, Lin Zhang, Tianxiang Luo

**Affiliations:** 1 Key Laboratory of Alpine Ecology, Institute of Tibetan Plateau Research, Chinese Academy of Sciences, Building 3, 16 Lin Cui Rd., Chaoyang District, Beijing, 100101 China; 2 CAS Center for Excellence in Tibetan Plateau Earth Sciences, Beijing, 100101 China

**Keywords:** Climate-change ecology, Grassland ecology, Climate-change impacts

## Abstract

It is still debatable whether temperature or precipitation mainly triggers spring vegetation green-up (SVG) in alpine dry ecosystems on the Tibetan Plateau. As phenological sensitivity to the arrival of monsoon-season rainfall would allow plants to simultaneously avoid drought and frost damages in the early growing season, we hypothesize that rainy season onset (RSO) rather than temperature mainly drives the spatiotemporal variability of SVG across alpine dry ecosystems over the Tibetan Plateau. Dates of RSO and SVG across 67 target areas nearby 67 weather stations over the Tibetan Plateau were calculated from time-series data of daily mean temperature and precipitation (1974–2013) and of the Normalized Difference Vegetation Index from the Moderate Resolution Imaging Spectroradiometer (2001–2013), respectively. Satellite-derived SVG was validated by 7-year observations (2007–2013) for leaf emergence of dominant species in alpine meadows along elevations (4400–5200 m) in Damxung of Tibet. We found that SVG generally synchronized with or was somewhat later than RSO although seasonal air temperatures were already continuously above 0 °C in 1 month before SVG dates. In pooled data across sites and years, the analysis of linear mixed model indicated that RSO (F = 42.109) and its interactions with pre-SVG precipitation (F = 6.767) and temperature (F = 4.449) mainly explained the spatio-temporal variability of SVG, while pre-SVG temperature and its interaction with precipitation did not have significant effects on SVG. Our data supported the hypothesis, suggesting that synchronization of SVG and RSO is a general spring phenological strategy across alpine dry ecosystems under influence of monsoon climate.

## Introduction

Identifying correct abiotic factors that initiate plant growth is crucial for understanding responses of species and ecosystems to climate change^1–5^. It has been suggested that the timing of leaf emergence in spring is highly sensitive to temperature variability^2,5,6^. Thus, the onset date of spring vegetation green-up is expected to continuously advance with recent climate warming, as generally observed in moist temperate and boreal ecosystems^6,7^. In arid and semi-arid ecosystems, however, variable and complex responses of spring phenology to climate warming have been observed on the Tibetan Plateau (delayed due to the reduction of chilling requirement^8^; unchanged with unclear driving factors^9,10^; advanced due to spring warming^11,12^; advanced due to the warming-induced increase of soil moisture^13^; advanced or unchanged due to interactions between snowfall and temperature^14^; delayed or advanced due to spatial variation of precipitation change and asymmetric impacts of daily maximum and minimum temperatures^15,16^; varied with data and methods^17^). Similar patterns are also found in other dry systems^5,7,18,19^. Recent experimental studies indicate that leaf emergence of dominant species in Tibetan alpine grasslands may vary little or even delay under experimental warming, but may be advanced by snow addition or watering^20,21^. Although it is well known that precipitation plays an important role in shaping species distribution and ecosystem function in dry regions, it is still unclear whether and how changes in precipitation regime such as rainy season onset may alter temperature effects on spring phenology. More recently, it has been observed that leaf emergence of dominant species in Tibetan alpine *Kobresia* meadows senses the arrival of monsoon-season rainfall^22^, and desert plants in Africa green up ahead of rainy season^23^. Because phenological responses to climate change may be involved in complicated evolutionary adaptations to environmental stresses^3,5,18,19,22,23^, it is challenging to understand climatic controls on spring vegetation phenology in dry regions, especially at high elevations where variable precipitation regimes and the interaction of temperature and precipitation may further confound the issue. In alpine dry ecosystems on the Tibetan Plateau, it is still debatable whether temperature or precipitation mainly drives the spatiotemporal variability of spring phenology though numerous studies have endeavored to search the possible cues^14,20–22^.

Plant phenology is a key component of ecological fitness, serving as an adaptive trait in shaping species distribution and ecosystem function^24^. On the Tibetan Plateau, the typical Indian monsoon circulation is established during late May and June when the plateau air temperature is generally above 0 °C, and the interaction of Indian monsoon and the westerlies generally creates a cold and dry climate in the pre-monsoon season^25^. The vast alpine meadows and steppes on the Tibetan Plateau are the zonal climax vegetation types adapted to the cold and dry climate, and their fan-shaped distribution pattern is mainly controlled by the warm and moist air of Indian summer monsoon through the atmospheric water transports along the high mountain valleys in eastern Himalayas^26^. The monsoon-westerly interaction may have selected genotypes that respond better to the pre-monsoon cold and dry climate. Our observed data from the *Kobresia* meadow in Damxung of Tibet suggest that phenological sensitivity to the arrival of monsoon-season rainfall would allow plants to simultaneously avoid drought and frost damages in the early growing season^22^. However, little attention has been paid to the effect of monsoon rainfall onset. Because of lack of long-term observed data in remote high mountains, few studies have investigated whether such a spring phenological strategy found in the *Kobresia* meadow may generally occur in different alpine vegetation types (alpine meadows, steppes, dry shrubs, etc.) over the Tibetan Plateau. To further test the generality of our previous findings in the *Kobresia* meadow^22^, we hypothesize that rainy season onset (RSO) rather than temperature mainly drives the spatiotemporal variability of spring vegetation green-up (SVG) across alpine dry ecosystems over the Tibetan Plateau. Such knowledge is important to understand the mechanisms underlying variable and complex responses of spring vegetation phenology to climate warming in the alpine dry region.

To test the hypothesis, dates of SVG and RSO across 67 target areas locating nearby 67 weather stations were calculated from the time-series data of daily mean temperature and precipitation (1974–2013) and the Normalized Difference Vegetation Index (NDVI) from the Moderate Resolution Imaging Spectroradiometer (MODIS, 2001–2013), respectively. First, we tested whether the satellite-derived SVG dates compare well to our 7-year observations (2007–2013) for leaf emergence of two dominant species in alpine meadows along elevations (4400–5200 m) in Damxung of Tibet. Second, we investigated whether SVG dates of 2001–2013 across vegetation types (alpine dry shrub, alpine meadow, and alpine steppe) generally synchronize with RSO dates although seasonal air temperatures are already continuously above 0 °C before SVG dates. Third, we examined whether there are similar patterns in interannual change trends of SVG and RSO across the 67 sites and among the three vegetation types. As earlier onset of Indian summer monsoon may result in earlier RSO and higher pre-monsoon precipitation^22,25^, we also examined whether SVG generally shows a positive correlation with RSO and a negative correlation with pre-SVG precipitation. Finally, we identified the key factor driving SVG by quantifying the relative effects of RSO and pre-monsoon temperature and precipitation and their interactions on the interannual variation of SVG across the 67 sites.

## Data and methods

### Target areas

By means of Google Earth and the Vegetation Atlas of China at Scale 1:1 Million^27^, 67 target areas (> 1000 pixels of 250 × 250 m MODIS image for each target area) locating nearby 67 weather stations (with a distance < 5 km) of China Meteorological Administration were selected to represent the typical vegetation types in arid and semi-arid zones of the Tibetan Plateau. The 67 target areas included 18 for alpine steppes, 33 for alpine meadows, and 16 for alpine dry shrubs. Each target area was selected on the same slope with an elevation difference of < 300 m relative to the weather station. Deserts and evergreen shrubs/woodlands were excluded in this study because the resolution of MODIS NDVI data is not enough to detect the small variation in seasonality of leaf area in desert plants and evergreen canopies^28^. To reduce the possible effects of irrigation and water level, croplands and wetlands were also excluded. Detailed information on geographical locations, vegetation types and related climate factors of the 67 target areas is found in Table S1.

### Satellite-derived SVG date and its validation

The time-series data of 16-day 250 m MODIS NDVI for the 67 target areas during 2001–2013 were obtained from the product of MOD13Q1 (MODIS/Terra Vegetation Indices 16-Day L3 Global 250 m SIN Grid, Version 5), freely available online at https://ladsweb.nascom.nasa.gov/data/. The MOD13Q1 product also includes a summary quality layer of pixel reliability, providing a quality assurance (QA) for each pixel in a certain period of time (0, ‘Good Data’; 1, ‘Marginal Data’; 2, ‘Snow/Ice’; 3, ‘Cloudy’). Across the 67 target areas, the average number of 16-day ‘Good Data’ images in the growing season (April to August) during 2001–2013 mainly ranged between 3 and 9, and increased with increasing latitude and longitude but varied little with elevation (Fig. S1, Table S2). Using the pixel-specific QA data, we conducted a data preprocessing procedure to remove the noises of snow cover and clouds according to the commonly accepted methods in the literature^15,29–32^. In alpine meadows and steppes, there is short-time snow cover in the non-growing season (November to March), which often reduces NDVI values and then leads to errors in retrievals of spring phenology^30,33^. To remove the snow-cover effect on NDVI, we first calculated the uncontaminated winter NDVI for each pixel as the mean value of 75–95% quantile of the uncontaminated winter NDVI values (flagged as ‘Good Data’) during November to March^15,31,32^. The contaminated winter NDVI value (flagged as ‘Snow/Ice’ or ‘Cloudy’) that is lower than the uncontaminated winter NDVI was substituted by the latter^30^. Clouds and poor atmospheric conditions generally depress NDVI values, which may result in an abrupt drop of NDVI in the growing season^29^. When the NDVI data were not flagged as ‘Good Data’, the abrupt drops of NDVI values during April and August (prior to the occurrence of maximum NDVI) were reconstructed by using the method of Savitzky-Golay filter^29^. To remove the noises of bare land and rock fields with sparse vegetation, each pixel for calculation should meet the following 3 criteria^15,16,32^: (1) the average NDVI during June and September should be higher than 0.10; (2) the annual maximum NDVI should be higher than 0.15 and occur in the growing season of July to September; (3) the average NDVI of July–September should be 1.2 times higher than that of November–March.

The preprocessed data of time-series NDVI were used for calculating SVG dates according to the relative threshold method of NDVI ratio developed by White, Thornton and Running^28^. The NDVI ratio (i.e., the ratio of NDVI change to annual maximum amplitude) was calculated as: NDVI_ratio_ = (NDVI_t_ − NDVI_min_)/(NDVI_max_ − NDVI_min_) × 100%. NDVI_t_ is the NDVI value at a certain time t; NDVI_max_ and NDVI_min_ are the maximum and minimum NDVI values, respectively, during the annual NDVI cycle. The SVG date for each year and each site was defined as the date when a NDVI ratio reached a threshold of 20%, as indicated in previous research on the Tibetan Plateau^8^.

To validate the methods used in this study, we tested the extent to which the satellite-derived SVG dates are consistent with our 7-year observations (2007–2013) for leaf emergence of two dominant species (*Kobresia pygmaea* sedge at 4650–5200 m; *Stipa capillacea* grass at 4400–4500 m) in alpine meadows along a large elevation gradient in Damxung of Tibet. The observed dataset was obtained from our recent report^22^.

### RSO date and other climate factors

Time-series data of daily mean temperature and precipitation across the 67 weather stations during 1974–2013 were obtained from China Meteorological Administration (https://cdc.nmic.cn/home.do). In each of the 67 weather stations, the RSO dates during 1974–2013 were calculated from the time-series data of daily mean temperature and precipitation. There is still no consensus on the most appropriate definition of rainy season onset^23,34^. In previous studies, rainy season onset is usually determined by using a variety of empirical precipitation thresholds which depend on the growth requirements of plants under different climatic conditions^23,35–37^. In this study, the RSO date was defined as the first day when a 5-days moving-average daily rainfall was higher than a constant threshold value, in which case the daily mean temperature was continuously above 0 °C^22^. By using different rainfall thresholds (ranging from 1 to 5 mm by a step of 0.5 mm) within a weather station, we calculated the correlation coefficients between satellite-derived SVG dates and the estimates of rainfall onset during 2001–2013. According to the highest correlation coefficient, the optimum rainfall threshold was determined for a weather station and then was used for calculating its RSO dates during 1974–2013. In this way, the interannual variation of RSO dates was independent of the constant, optimum rainfall threshold used for a weather station. Because of the geographical variations in rainfall intensity and plant growth requirements, the estimated optimum rainfall thresholds varied with study sites and vegetation types (Fig. S2a), ranging from 3.2 to 3.5 mm in steppes and meadows to 4.0 mm in shrubs (Fig. S2b).

To further explore ecologically meaningful, interannually comparable climate factors driving the change of SVG, the mean temperature and precipitation of 30 or 60 days before a multi-year mean SVG date (T_-30d_ and Pr_-30d_ or T_-60d_ and Pr_-30d_; so called pre-SVG temperature and precipitation) were calculated as in Li et al*.*^22^.

### Data analysis

Simple linear regression was used for testing the interannual change trends in SVG, RSO and pre-SVG temperature (T_-30d_, T_-60d_) and precipitation (Pr_-30d_, Pr_-60d_) as well as their relationships to each other. At site level and in grouped data for shrubs (16 sites), meadows (33 sites) and steppes (18 sites), partial correlation analysis of multiple linear regressions was used for assessing the relative importance of RSO, T_-30d_ and Pr_-30d_ in determining the interannual variation of SVG during 2001–2013. In pooled data across the 67 sites during 2001–2013, the analysis of variance with Type III sum of squares under the framework of linear mixed model was used for quantifying the relative effects of RSO, T_-30d_, Pr_-30d_ and their interactions on the variation of SVG, in which the station ID was taken as the subject; RSO, T_-30d_, Pr_-30d_ and their interactions were taken as fixed factors, while random effects of RSO, T_-30d_ and Pr_-30d_ were considered.

All statistical analyses were performed using SPSS 18.0 (SPSS Inc., Chicago, Illinois, USA) and OriginPro 9.0 for Windows (OriginLab Corporation, Northampton, USA). All the maps were drawn by ArcGIS 10.3 for Desktop (Environmental Systems Research Institute, Inc., RedLands, California, USA). All significant correlations were taken at P < 0.05.

## Results

### Comparison between satellite-derived SVG and ground observations

**Figure 1:**
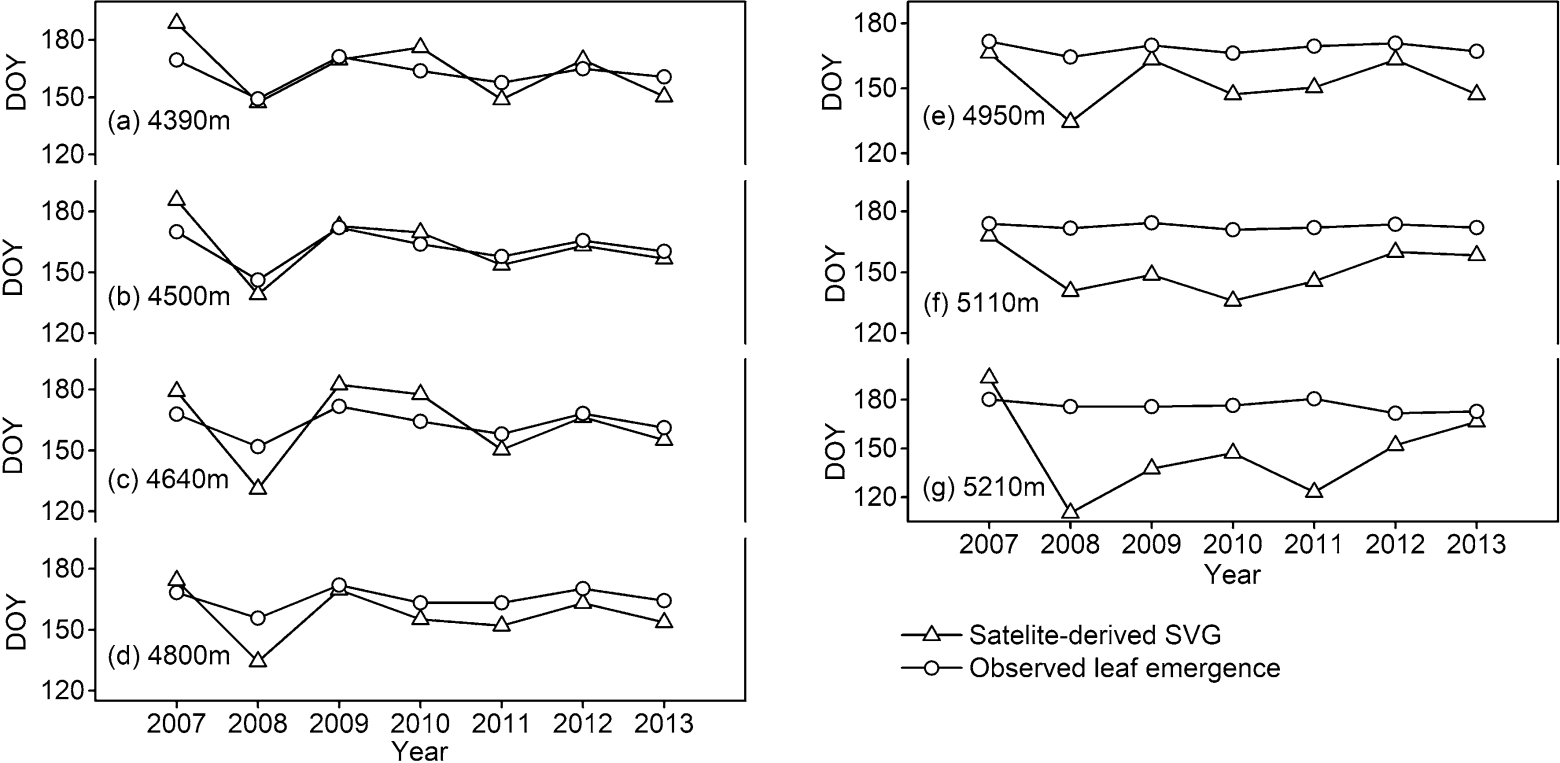
Interannual variations of satellite-derived spring vegetation green-up (SVG) dates were compared to the observed leaf emergence dates of two dominant species (*Kobresia pygmaea* sedge at 4650–5200 m; *Stipa capillacea* grass at 4400–4500 m) along a large elevation gradient in Damxung of Tibet during 2007–2013.

The satellite-derived SVG dates synchronized well with the 7-year observations of leaf emergence dates (2007–2013) for two dominant species of alpine meadows along elevations from 4400 to 4800 m (Fig. 1a–d; R = 0.90, P < 0.05), but not at higher elevations close to the upper limit of *Kobresia* meadows (4950–5200 m, Fig. 1e–g). As the elevations of all the 67 target areas were less than 4800 m (Table S1), our observed data validated the methods for satellite-derived SVG dates at elevations below 4800 m on the Tibetan Plateau.

### Relationships of SVG to RSO and pre-SVG temperature and precipitation

**Figure 2:**
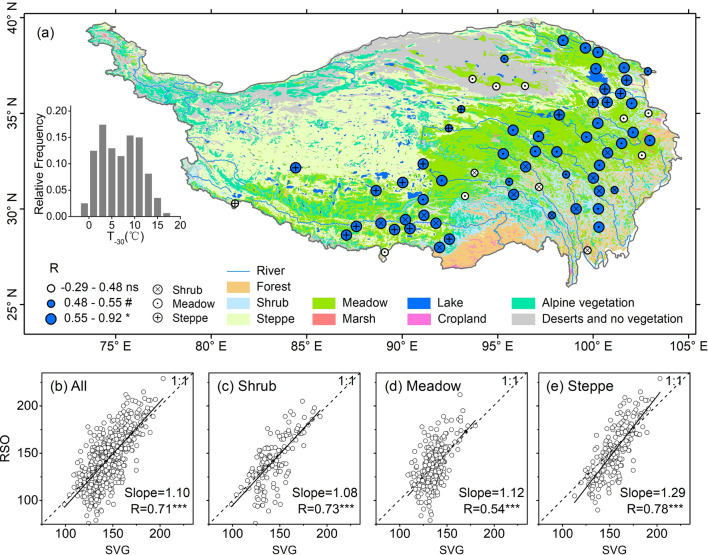
Relationships between interannual variations of spring vegetation green-up (SVG) and rainy season onset (RSO) across 67 sites during 2001–2013. (**a**) Spatial variability of correlation coefficients (R) between SVG and RSO dates during 2001–2013 for each of the 67 stations; different sizes of the circles indicate significant levels of the correlation coefficients (*P < 0.05; ^#^P < 0.10; ns, not significant); the background is the distribution map of vegetation types, which was drawn using ArcGIS 10.3 for Desktop with the vector data for Vegetation Atlas of China (1:1,000,000)^27^ being available free online at https://www.geodata.cn/; the left inset shows the frequency distribution of pre-SVG mean temperature (T_-30d_) among the 67 sites. (**b**–**e**) Correlations between RSO and SVG dates during 2001–2013 in pooled data of the 67 sites (**b**) and in grouped data for alpine dry shrubs (**c**), alpine meadows (**d**) and alpine steppes (**e**), respectively; the 1:1 dashed line indicates the one-to-one relationship; ^***^P < 0.001.

The pre-SVG mean temperature (T_-30d_) across the 67 sites was typically above 0 °C in 1 month before SVG dates (ranging between 1 and 16 °C in 65 of the 67 sites; inset in Fig. 2a). At site level, the positive correlation between SVG and RSO during 2001–2013 was observed in 55 of the 67 sites (P < 0.05 for 47 sites, P < 0.10 for 8 sites; Fig. 2a). An almost one-to-one relationship between SVG and RSO during 2001–2013 was found in pooled data across the 67 sites (Fig. 2b, slope = 1.10) and in grouped data for shrubs (Fig. 2c, slope = 1.08) and meadows (Fig. 2d, slope = 1.12). In alpine steppes, SVG was somewhat later than RSO (Fig. 2e, slope = 1.29).

**Figure 3:**
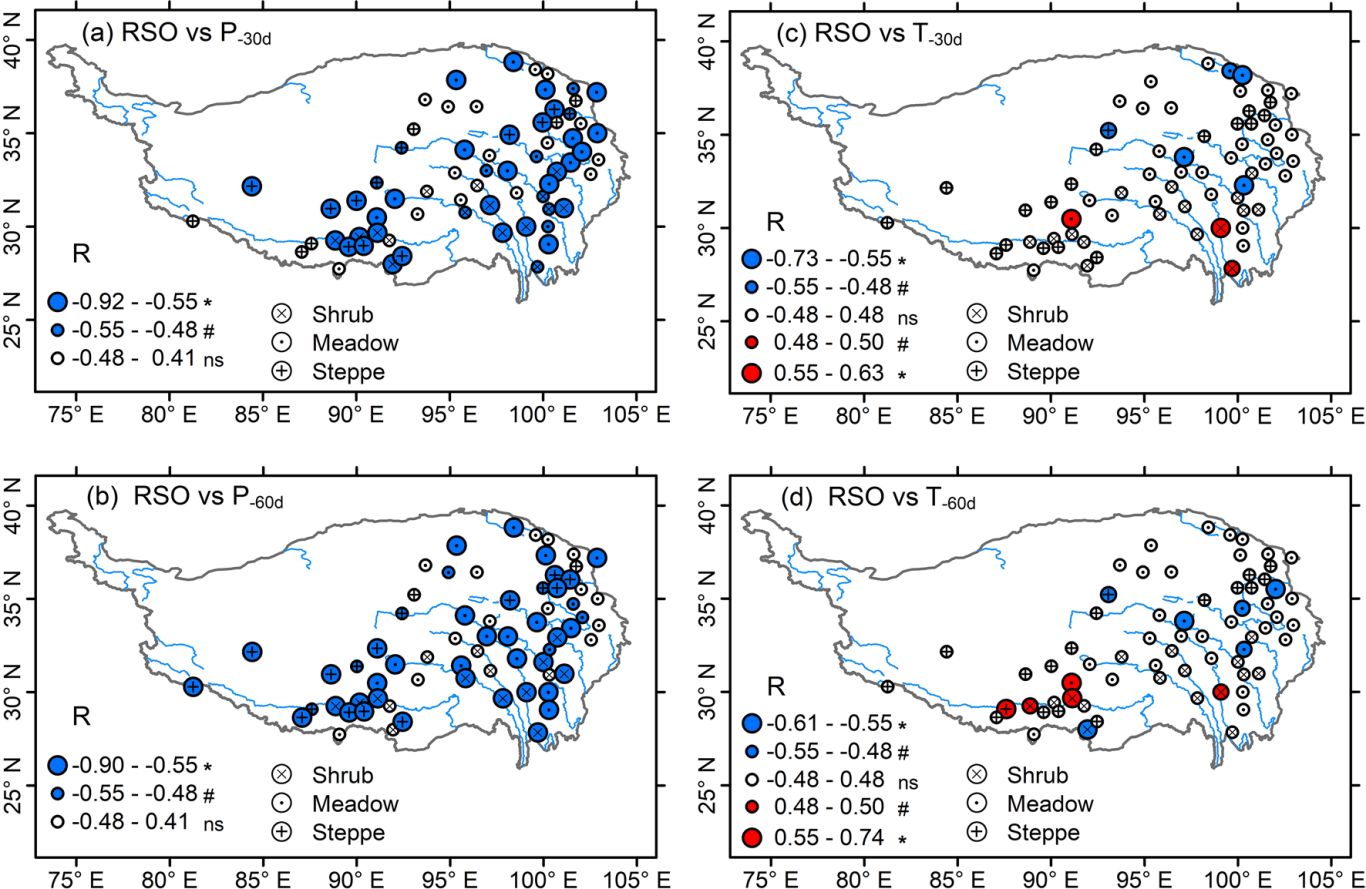
Correlation coefficients (R-value) between rainy season onset (RSO) and pre-SVG precipitation (P_-30d_, P_-60d_) (**a**,**b**) and mean temperature (T_-30d_, T_-60d_) (**c**,**d**) for each of the 67 sites during 2001–2013. Different sizes of the circles indicate the significant levels of correlation coefficients (*P < 0.05; ^#^P < 0.10; ns, not significant). The map was drawn with the vector data available free online at https://www.geodata.cn/, using ArcGIS 10.3 for Desktop.

**Figure 4:**
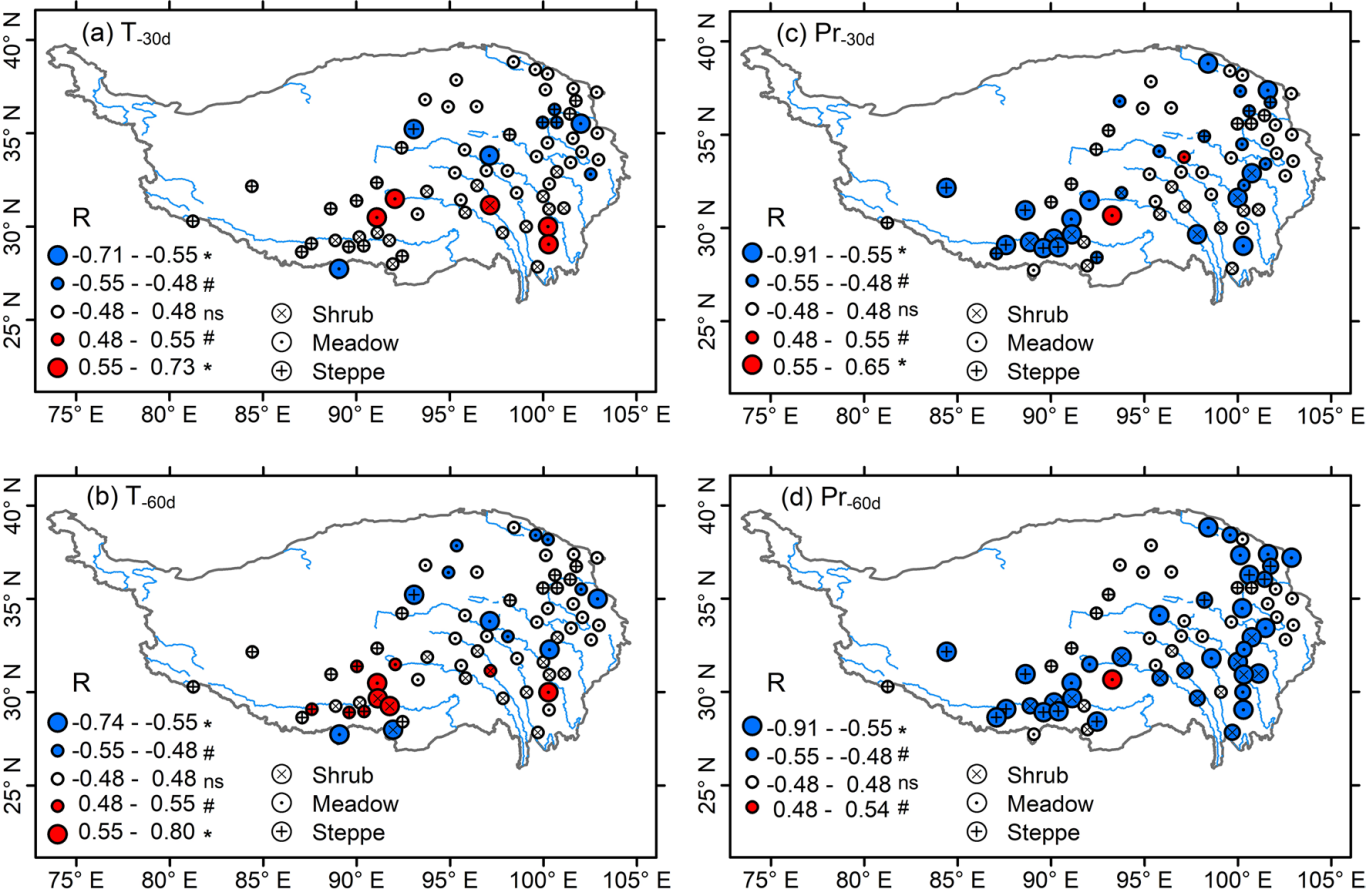
Relationships of SVG date to (**a**,**b**) pre-SVG mean temperature (T_-30d_, T_-60d_) and (**c**,**d**) pre-SVG precipitation (P_-30d_, P_-60d_) for each of the 67 sites during 2001–2013. Different sizes of the circles indicate the significant levels of correlation coefficients (*P < 0.05; ^#^P < 0.10; ns, not significant). Red circles, SVG delayed with increasing temperature (precipitation). Blue circles, SVG advanced with increasing temperature (precipitation). The map was drawn with the vector data available free online at https://www.geodata.cn/, using ArcGIS 10.3 for Desktop.

During 2001–2013, T_-30d_ had an increasing trend in 28 of the 67 sites (P < 0.05 for 17 sites, P < 0.10 for 11 sites) but varied little in remaining other sites (Fig. S3a). Pre-SVG precipitation (Pr_-30d_) varied little in most cases (except 5 sites with a significant decreasing or increasing trend; Fig. S3c). RSO dates were negatively correlated with pre-SVG precipitation (Pr_-30d_, Pr_-60d_) (Fig. 3a,b, P < 0.05 for 32/37 sites, P < 0.10 for 11/8 sites), but varied little with pre-SVG mean temperatures in most cases (Fig. 3c,d, except for 8–11 of the 67 sites where there were positive or negative correlations between RSO and T_-30d_/T_-60d_). Thus, SVG dates of 2001–2013 showed a general negative correlation to pre-SVG precipitation in almost half of the 67 sites (Fig. 4c,d). In comparison, SVG dates were not correlated with pre-SVG mean temperatures (T_-30d_, T_-60d_) in most cases, only in 9–10 of the 67 sites where there were positive or negative correlations between SVG and T_-30d_ (T_-60d_) (Fig. 4a,b).

**Figure 5:**
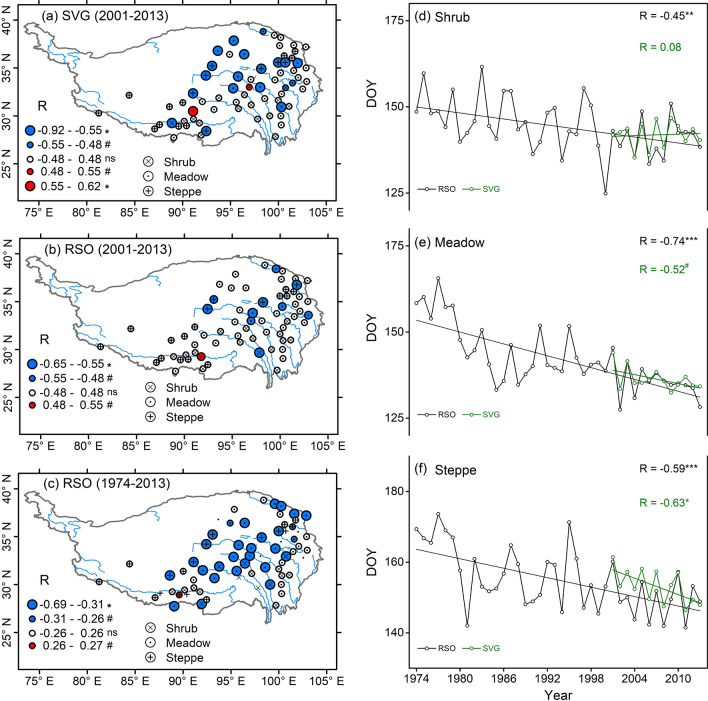
Interannual change trends in (**a**) satellite-derived SVG dates during 2001–2013 and (**b**,**c**) RSO dates during 2001–2013 and 1974–2013 for each of the 67 sites, and (**d**–**f**) the advancing trends in mean RSO and SVG dates grouped for alpine dry shrubs (**d**), alpine meadows (**e**), and alpine steppes (**f**). (**a**–**c**) R is the statistical value for a linear trend; different sizes of the circles indicate the significant levels of R-values (*P < 0.05; ^#^P < 0.10; ns, not significant); blue cycles, advanced trends of SVG and RSO; red circles, delayed trends of SVG and RSO. (**d**–**f**) Significant trend lines: *P < 0.05, ***P < 0.001; the slopes of RSO and SVG trend lines (i.e. advanced days per 10 years): 2.9 (P < 0.001) and 0.7 (not significant) for alpine shrubs, 5.7 (P < 0.001) and 4.3 (P < 0.05) for alpine meadows, 4.5 (P < 0.001) and 7.8 (P < 0.05) for alpine steppes. The map was drawn with the vector data available free online at https://www.geodata.cn/, using ArcGIS 10.3 for Desktop.

SVG dates of 2001–2013 showed an advancing trend in 19 of the 67 sites (P < 0.05 for 16 sites, P < 0.10 for 3 sites) but typically varied little in remaining other sites (except one site with a significant delaying trend; Fig. 5a). RSO showed an advancing trend in 10 of the 67 sites during 2001–2013 (Fig. 5b, P < 0.05 for 5 sites, P < 0.10 for 5 sites) and in 29 of the 67 sites during 1974–2013 (Fig. 5c, P < 0.05 for 26 sites, P < 0.10 for 3 sites), but varied little in remaining other sites (Fig. 5b,c). In grouped data for shrubs, meadows and steppes, mean RSO dates generally showed an advancing trend during 1974–2013 (P < 0.001, Fig. 5d–f). During 2001–2013, mean RSO and SVG dates both advanced significantly in meadows and steppes but varied little in shrubs (Fig. 5d–f), in which SVG dates synchronized with RSO dates in meadows and shrubs (Fig. 5d,e) and were somewhat later than RSO dates in steppes (Fig. 5f).

### Relative effects of RSO and other climate factors on interannual variation of SVG

Partial correlation analysis indicated that RSO had the highest significant effect on SVG in grouped data for each of the 3 vegetation types (R = 0.52–0.73, P < 0.001), compared to lower or insignificant effects of T_-30d_ and Pr_-30d_ (Table 1; the highest effect of RSO on SVG was also found in site-level data, Table S3).

**Table 1:** Partial correlation coefficients of multiple regressions for relationship of SVG to RSO, T_-30d_ and P_-30d_ in grouped data for shrubs (16 sites), meadows (33 sites) and steppes (18 sites) and in pooled data across the 67 sites during 2001–2013.

Vegetation types	RSO	T_-30d_	P_-30d_
Alpine dry shrubs (n = 208)	0.52***	0.47***	− 0.23***
Alpine meadows (n = 429)	0.54***	0.40***	0.02
Alpine steppes (n = 234)	0.73***	− 0.03	0.11
Total (n = 871)	0.64***	0.28***	− 0.03

***P < 0.001.

In pooled data across the 67 sites during 2001–2013, the analysis of variance in linear mixed model indicated that RSO had the highest significant effect on SVG (F = 42.109, P < 0.001), being much higher than the significant effects of Pr_-30d_ (F = 5.403, P = 0.021), RSO × Pr_-30d_ (F = 6.767, P = 0.010) and RSO × T_-30d_ (F = 4.449, P = 0.035) (Table 2). T_-30d_, T_-30d_ × Pr_-30d_ and RSO × T_-30d_ × Pr_-30d_ did not have significant effects on SVG (Table 2).

**Table 2:** Summary of Type III tests of linear mixed model for fixed effects of RSO, T_-30d_, Pr_-30d_ and their interactions on the variation of SVG in pooled data across the 67 sites during 2001–2013.

Source	Numerator df	Denominator df	*F*	*P*
Intercept	1	459.536	156.542	< 0.001
RSO	1	673.096	42.109	< 0.001
T_-30d_	1	538.181	3.459	0.063
Pr_-30d_	1	479.065	5.403	0.021
RSO × T_-30d_	1	691.966	4.449	0.035
RSO × Pr_-30d_	1	454.909	6.767	0.010
T_-30d_ × Pr_-30d_	1	527.574	1.631	0.202
RSO × T_-30d_ × Pr_-30d_	1	497.179	0.617	0.433

The fixed effects were tested by using analysis of variance with Type III sum of squares under the framework of linear mixed model. Denominator df is equal to within-group sum of squares.

## Discussion

The Tibetan Plateau, which is known as the roof of the world, is one of the regions with strongest warming^38^. In recent decades, the whole plateau has become warmer and wetter^39^, which is expected to have a positive effect on the world’s highest and largest alpine vegetation (meadows, steppes, dry shrubs, etc.)^40–42^. In response to the recent warming and wetting, however, there are variable trends (advanced, delayed, unchanged) in satellite-derived green-up dates^8,11,12,15–17^ and long-term observed leaf emergence of dominant species^9,10,14^. In this study, our data indicated that SVG dates across alpine dry ecosystems generally synchronized with or were somewhat later than RSO dates although seasonal air temperatures were already continuously above 0 °C in 1 month before SVG dates (Figs. 2 and 5). Earlier RSO and higher pre-monsoon precipitation are generally linked to earlier onset of Indian summer monsoon^22,25^, which explains why earlier RSO dates typically resulted in higher pre-SVG precipitation (Fig. 3a,b) regardless of pre-SVG temperature (Fig. 3c,d). Accordingly, SVG dates showed a general negative correlation with pre-SVG precipitation (Fig. 4c,d) but did not correlate with pre-SVG temperature in most cases (Fig. 4a,b). There were similar patterns in change trends of RSO and SVG across the 67 sites and among the three vegetation types (Fig. 5). The analysis of linear mixed model further indicated that RSO and its interactions with pre-SVG temperature and precipitation mainly explained the spatio-temporal variability of SVG dates across arid and semi-arid ecosystems on the Tibetan Plateau, while pre-SVG temperature and its interaction with pre-SVG precipitation did not have significant effects on SVG (Table 2). The findings suggest that the cold and dry climate in the pre-monsoon season is one of main filters for the survival and distribution of dominant species in alpine dry ecosystems, and phenological sensitivity to rainy season onset allows plants to simultaneously avoid drought and frost damages in the early growing season. Such a mechanism provides a general explanation for the spatially variable greening responses to climate change in the vast alpine grasslands and shrublands.

Since the arrival of monsoon rainfall mainly depends on the onset and intensity of Indian summer monsoon circulation and the complex effects of topography^43^, large spatial and temporal variations in RSO and SVG dates are expected (Figs. 3, 4, 5). In some cases, there were spatially positive or negative correlations between SVG and pre-SVG temperature (Fig. 4a,b) as reported in previous studies^14,32^, which would be mainly caused by the positive/negative correlations between RSO and pre-SVG temperature (Fig. 3c,d). It has been suggested that Indian summer monsoon is weakening and the westerlies are reinforcing since the 1970s, resulting in decreased precipitation in the Himalayas but increased precipitation in northern Tibetan Plateau^44^. In southern Tibetan Plateau, the weakened monsoon may lead to later onset of RSO and thus less precipitation and higher temperature (due to decreased cloud cover and increased solar radiation) in the pre-monsoon season, which explains the positive correlation between RSO and pre-SGV temperature (Fig. 3c,d) and the negative correlation between pre-SVG precipitation and temperature (Fig. S4a,b) at some sites in the southern part. In northern Tibetan Plateau, the enhanced westerlies may lead to increased spring snowfall and the spring warming may indirectly induce earlier onsets of RSO and SVG by increasing snowmelt water (i.e., soil water availability^13^) and/or the ratio of rainfall to snowfall^22^, which explains the negative correlations of RSO and SVG to pre-SGV temperature at some sites in the northern part (Fig. 3c,d).

It should be noted that at high elevations close to the upper limit of the *Kobresia* meadows (4950–5200 m, Fig. 1e–g), satellite-derived SVG dates (DOY 110–170) were much earlier than the observed leaf emergence dates (DOY 170–180), compared to the significant consistency between satellite-derived and observed values at lower elevations (4400–4800 m, Fig. 1a–d). In the early growing season (April to June) when precipitation often occurs at night time, it often snows and sleets at high elevations above 4800 m but rains at lower elevations. While the snowfall has no effect on plant growth, the early-season snow melting may increase the NDVI values and then result in incorrect estimation of SVG dates derived from satellite data at elevations above 4800 m. In this study, such uncertainty should be minor because the elevations of all the 67 target areas were less than 4800 m (Table S1). In the future, field experimental data are needed to verify the optimum rainfall threshold for RSO and the NDVI ratio threshold for SVG.

Our observed data in Damxung of Tibet further indicated that leaf emergence of dominant species in the alpine meadows was highly sensitive to the arrival of monsoon rainfall but insensitive to that of snowfall^22^, resulting in later green-up dates above 4800 m than at lower elevations (Fig. 1). At the upper limit of the *Kobresia* meadows (5100–5200 m, Fig. 1f,g), the observed green-up dates varied little during 2007–2013 and typically occurred at the end of June (DOY 180) when the daily mean air-temperature was continuously above 0 °C, setting a threshold of minimum growing days that allows plants to have enough time to safely complete their life cycle. This is consistent with our previous reports that the number of days with daily mean soil temperature ≥ 5 °C (at − 10 cm) during June and August is about 80–90 days at 5100–5200 m^45^, with a seasonal mean soil temperature of 6.8–7.3 ℃^46^ being close to the low temperature threshold that significantly limits treeline growth and photosynthesis^47–50^.

The ratio of rainfall to snowfall usually decreases with increasing elevation. Spring warming may increase the rainfall fraction and then indirectly advance the green-up dates of alpine plants at high elevations. This is typically consistent with some previous observations on the spring phenological response to precipitation change in this region. Using observed data from 23 phenological stations on the Tibetan Plateau, Chen et al.^14^ indicated that the different interactions between snowfall and temperature during late winter and early spring likely determine the spatiotemporal variations of green-up dates. In the central Himalaya, Pangtey et al.^51^ found that the growth initiation of 184 alpine plant species generally synchronizes with the beginning of snow melt period.

In conclusion, our data supported the hypothesis, indicating that monsoon rainfall onset and its interactions with pre-monsoon temperature and precipitation mainly drives the spatio-temporal variability of spring green-up date across alpine dry ecosystems over the Tibetan Plateau. Our data further suggest that synchronization of spring vegetation green-up and rainy season onset is a general spring phenological adaptation to the pre-monsoon cold and dry climate. The threshold of minimum growing days determining the upper limit of alpine meadows/steppes would be linked to the sensitivity of spring phenology to the arrival of monsoon-season rainfall. Long-term observations and experiments, which are very scarce in remote high mountains, are required to verify the findings based on satellite-derived data.

## Supplementary information

Supplementary Information.
