# Supplementary material for: Rainy season onset mainly drives the spatiotemporal variability of spring vegetation green-up across alpine dry ecosystems on the Tibetan Plateau
Source: Sci Rep. 2020 Nov 2;10:18797. doi: 10.1038/s41598-020-75991-w (PMC7606468; doi:10.1038/s41598-020-75991-w)
Supplement: Supplementary file 1 — Supplementary Information. [file 41598_2020_75991_MOESM1_ESM.pdf]

supplementary Information for

Rainy season onset mainly drives the spatiotemporal variability of spring vegetation green-up across alpine dry ecosystems on the Tibetan Plateau

Xiang Li[1], Lin Zhang[1,2], Tianxiang Luo[1,2*]

[1]Key Laboratory of Alpine Ecology, Institute of Tibetan Plateau Research, Chinese Academy of Sciences, Beijing, 100101, China

[2]CAS Center for Excellence in Tibetan Plateau Earth Sciences, Beijing 100101, China

***Corresponding Author:**

Dr. Tianxiang Luo, Professor in ecology and biogeography

Key Laboratory of Alpine Ecology, Institute of Tibetan Plateau Research, Chinese Academy of Sciences; Building 3, 16 Lin Cui Rd., Chaoyang District, Beijing 100101, China; Fax: +86 10 84097060; Email: luotx@itpcas.ac.cn

Contents of this file:

Tables S1-S3

Figures S1-S4

Table S1. List of geographical locations, vegetation types, annual mean temperature and precipitation, mean RSO and SVG dates across 67 target areas on the Tibetan Plateau

Weather stations	Latitude (°)	Longitude (°)	Vegetation types	Altitude (m)	Pixel count	MAT (℃)	MAP (mm)	Mean RSO (DOY)	Mean SVG (DOY)
Tuole	38.82	98.42	Meadow	3367	2649	-1.6±0.1	333.2±11.3	158.7±5.8	149.3±3
Yeniugou	38.42	99.58	Meadow	3320	1731	-2.1±0.1	460.4±15.7	140±5.3	142.7±1.7
Qilian	38.18	100.25	Meadow	2787	2202	2±0.1	416±17.4	143.2±5.5	140.1±1.5
Xiaozaohuo	36.80	93.68	Meadow	2767	569	5.1±0.1	33±4.3	157.2±9.4	157.6±2.7
Dachaidan	37.85	95.35	Meadow	3173	1421	3.2±0.1	104.5±9.6	148.4±7.4	143±2.1
Gangcha	37.33	100.13	Meadow	3302	2762	0.6±0.1	392.8±11.2	138.2±4	140.9±1.7
Menyuan	37.38	101.62	Meadow	2850	521	1.9±0.1	517.8±17.3	128.8±5.7	125±1.3
Wushaoling	37.20	102.87	Meadow	3045	2333	0.8±0.1	420.4±21.9	126.1±2.8	132±1.3
Golmud	36.42	94.92	Meadow	2808	1412	6.5±0.1	46.2±5.3	138.8±7.5	144±1.7
Nomhon	36.43	96.43	Meadow	2790	1760	6±0.1	56.1±8.4	147.7±6.1	159.6±2.1
Gonghe	36.27	100.62	Steppe	2835	3758	5.3±0.1	328.2±8.4	146.5±6	143.3±3.4
Xining	36.73	101.75	Steppe	2295	3490	6±0.1	431.2±15.7	126.9±4.5	132±2.4

Guide	36.03	101.43	Steppe	2237	2314	8.1±0.1	265.2±12.6	132.4±5.9	136.7±4.4
Wudaoliang	35.22	93.08	Steppe	4612	4911	-4.4±0.1	341.3±17.3	148.2±1.6	152.8±2.5
Xinghai	35.58	99.98	Steppe	3323	3242	2±0.1	403.4±20.2	114±4	146.3±3.5
Guinan	35.58	100.73	Steppe	3120	2642	2.9±0.1	445.4±19.6	127.2±4.8	140.6±3.2
Tongren	35.52	102.02	Meadow	2491	2288	6.9±0.1	423.2±18.4	119.6±3	126.2±1.7
Gerze	32.15	84.42	Steppe	4415	4951	1.3±0.2	206.4±10.8	167.4±6.8	168±2.7
Baingoin	31.38	90.02	Steppe	4700	2743	0.3±0.1	357.6±22.5	165.4±4.9	153.1±1.7
Amdo	32.35	91.10	Steppe	4800	2232	-1.7±0.1	491.8±19.9	151.2±1.9	155.7±2
Nagqu	31.48	92.07	Meadow	4507	4693	0.1±0.1	467.3±23.7	142.5±4.4	145.3±2
Purang	30.28	81.25	Steppe	3900	186	4.3±0.1	156.3±15.4	123.7±10.7	119.2±2
Shenza	30.95	88.63	Steppe	4672	1891	0.8±0.1	379.8±17.9	154.8±5.3	160.7±3
Damxung	30.48	91.10	Meadow	4200	1585	2.6±0.1	497.2±33.4	149.3±5.4	152.7±3.7
Lhatse	29.08	87.60	Steppe	4000	1535	7.6±0.1	332.5±21	174.3±6.2	166±3
Shigatse	29.25	88.88	Shrub	3836	1746	7.3±0.1	418.6±23.2	164.4±5.8	165.1±3.8
Nyêmo	29.43	90.17	Shrub	3809	1550	7.6±0.1	349.1±17.8	157.5±4.8	158.8±4.2
Lhasa	29.67	91.13	Shrub	3649	1415	9.3±0.2	464.8±24.1	160.3±6.6	151.9±3.8
Zetang	29.25	91.77	Shrub	3552	1630	9.6±0.2	396.4±28.4	155.2±6.3	155.1±3.7

Tingri	28.63	87.08	Steppe	4300	2684	3.8±0.1	297±21.8	167.3±6.1	174.4±3.2
Gyantse	28.92	89.60	Steppe	4040	2137	5.7±0.1	275.1±17.2	178.3±7.2	172.4±3.9
Nagarze	28.97	90.40	Steppe	4432	1462	3.7±0.1	393.1±25.6	172.2±7.6	170.8±3.5
Cona	27.98	91.95	Shrub	4280	2877	0.6±0.1	422±12.7	117.2±2.9	117.8±2.6
Lhunze	28.42	92.47	Steppe	3860	1367	5.9±0.1	297.7±18.7	179.8±6.5	162.4±2.4
Pagri	27.73	89.08	Meadow	4302	3497	0.9±0.1	446.5±19.2	111.2±4.1	129.4±1.5
Tuotuo-he	34.22	92.43	Steppe	4533	3518	-2.8±0.1	339.5±20	148.5±3	151.1±1.7
Zadoi	32.88	95.28	Meadow	4066	1846	1.8±0.1	542.6±20.9	123.7±2.6	139±1
Qumalai	34.12	95.80	Meadow	4175	2332	-0.9±0.1	465.2±19.4	134.9±3.7	140.8±2.1
Yushu	33.00	96.97	Meadow	3681	1867	4.4±0.2	485.3±23.2	129.2±4.4	136.7±1.3
Madoi	34.92	98.22	Steppe	4272	3042	-2.6±0.1	351.2±17.6	137.8±3.3	151±2.3
Qingshui-he	33.80	97.13	Meadow	4415	4280	-3.5±0.2	544.3±20.9	167.1±8.3	140.9±1.4
Shiqu	32.98	98.10	Meadow	4200	2402	-0.2±0.1	571±30.8	133.3±2.7	130.7±1.6
Maqin	34.48	100.23	Meadow	3719	2924	0.6±0.1	529±21.6	139.2±4.5	134.9±1.2
Dari	33.75	99.65	Meadow	3968	2652	0.1±0.1	574.6±19.9	118.3±2.6	136.9±1.7
Henan	34.73	101.60	Meadow	3500	3824	0.4±0.1	587.9±26.7	127.1±3.3	126.4±1.6
Jigzhi	33.43	101.48	Meadow	3629	2669	1.7±0.1	733.1±26.4	131.8±3.5	127±1.5

Maqu	34.00	102.08	Meadow	3471	3002	2.6±0.1	610.8±19.2	133.5±3	124.7±2.1
Zoige	33.58	102.97	Meadow	3440	4821	2.2±0.1	649.9±31.1	139.5±5.1	116.9±1.4
Hezuo	35.00	102.90	Meadow	2910	3628	3.3±0.1	548.8±22	121.7±4.4	123.9±1.5
Sog	31.88	93.78	Shrub	4023	1774	2.9±0.1	633.2±23.1	134.4±2.3	141±1.1
Dênqên	31.42	95.60	Meadow	3873	2232	4.2±0.1	657.5±24.5	128.4±7	131.7±0.8
Nangqên	32.20	96.47	Shrub	3644	2163	5.3±0.1	571.4±22.1	140.7±1.8	137.4±1.2
Qamdo	31.15	97.17	Shrub	3306	827	8.2±0.1	490.3±19.4	146.8±5.1	139.8±1.1
Dêgê	31.80	98.58	Meadow	3184	365	7.2±0.1	614.6±26.5	107.6±6.6	131.1±1.2
Garzê	31.62	100.00	Shrub	3394	1679	6.4±0.1	640.3±24.6	129.1±5	127.6±1.6
Baima	32.93	100.75	Shrub	3530	3530	3.6±0.1	639.4±23.2	134.8±2.9	131.6±1.8
Sêrtar	32.28	100.33	Meadow	3894	2318	1.1±0.1	659.8±29.7	137.6±4	132.4±1.6
Dawu	30.98	101.12	Shrub	2957	1094	8.4±0.1	588.9±25.3	134.2±6	131±1.5
Hongyuan	32.80	102.55	Meadow	3492	2233	2.3±0.1	723.3±32.1	101±3.3	112.7±1.5
Lhari	30.67	93.28	Meadow	4489	285	0.3±0.1	751.2±31.7	140.3±4.7	139.7±2
Lhorong	30.75	95.83	Shrub	3640	1745	6.2±0.1	414.5±19.4	133.5±8.6	136.5±2.1
Batang	30.00	99.10	Shrub	2589	1513	13.4±0.1	465.8±30.4	141.7±7.8	149.5±2.9
Xinlong	30.93	100.32	Shrub	3000	1608	8±0.1	651.3±23	117.1±4	133±1.3

Litang	30.00	100.27	Meadow	3949	4256	4.3±0.1	760.4±31.2	128.2±6.3	138.6±1.1
Zogang	29.67	97.83	Shrub	3780	646	5.2±0.1	456.5±21	143±7.5	153.9±2.4
Daocheng	29.05	100.30	Meadow	3728	3149	5.3±0.1	652.4±35.4	149.8±5.3	146.4±2.5
Shangri-la	27.83	99.70	Shrub	3276	1372	6.9±0.1	620.2±31.5	138.6±4.6	139.5±2.5

Table S2. The average number of 16-day 'Good Data' images (GDIs) in the growing season (April to August) and the non-growing season (November to March) for each of the 67 target areas during 2001-2013.

Weather stations	Latitude (°)	Longitude (°)	Vegetation types	Altitude (m)	GDIs of growing season	GDIs of non-growing season
Tuole	38.82	98.42	Meadow	3367	7.1±0.8	7.8±0.5
Yeniugou	38.42	99.58	Meadow	3320	7.2±0.5	6.4±0.6
Qilian	38.18	100.25	Meadow	2787	9.3±0.2	7.6±0.7
Xiaozaohuo	36.80	93.68	Meadow	2767	9.5±0.3	9.4±0.3
Dachaidan	37.85	95.35	Meadow	3173	2.2±0.5	2.9±0.5
Gangcha	37.33	100.13	Meadow	3302	6.8±0.4	6.2±0.5
Menyuan	37.38	101.62	Meadow	2850	8.8±0.4	6.8±0.4
Wushaoling	37.20	102.87	Meadow	3045	7.4±0.5	4.8±0.7
Golmud	36.42	94.92	Meadow	2808	0.7±0.2	2.8±0.7
Nomhon	36.43	96.43	Meadow	2790	4.3±0.6	3.8±0.6
Gonghe	36.27	100.62	Steppe	2835	2.7±0.5	4.9±0.6
Xining	36.73	101.75	Steppe	2295	4.7±0.5	3±0.5

Guide	36.03	101.43	Steppe	2237	5.1±0.5	6.5±0.4
Wudaoliang	35.22	93.08	Steppe	4612	8.5±0.4	8.9±0.3
Xinghai	35.58	99.98	Steppe	3323	9.2±0.2	9.7±0.2
Guinan	35.58	100.73	Steppe	3120	9.5±0.2	9.5±0.2
Tongren	35.52	102.02	Meadow	2491	6.8±0.4	8.2±0.6
Gerze	32.15	84.42	Steppe	4415	8.5±0.3	9.8±0.1
Baingoin	31.38	90.02	Steppe	4700	7.2±0.3	9.2±0.3
Amdo	32.35	91.10	Steppe	4800	8±0.4	9.8±0.2
Nagqu	31.48	92.07	Meadow	4507	1.6±0.3	7±0.5
Purang	30.28	81.25	Steppe	3900	5.9±0.4	6.8±0.4
Shenza	30.95	88.63	Steppe	4672	7.4±0.3	9.6±0.1
Damxung	30.48	91.10	Meadow	4200	7.8±0.2	9.6±0.2
Lhatse	29.08	87.60	Steppe	4000	3.8±0.6	10±0
Shigatse	29.25	88.88	Shrub	3836	3.1±0.3	9.5±0.2
Nyêmo	29.43	90.17	Shrub	3809	3.9±0.4	9.8±0.1
Lhasa	29.67	91.13	Shrub	3649	3.9±0.4	9.8±0.1
Zetang	29.25	91.77	Shrub	3552	7.2±0.5	9.9±0.1

Tingri	28.63	87.08	Steppe	4300	6.5±0.5	10±0
Gyantse	28.92	89.60	Steppe	4040	1.7±0.3	8.1±0.4
Nagarze	28.97	90.40	Steppe	4432	1.8±0.4	9.7±0.1
Cona	27.98	91.95	Shrub	4280	1.1±0.3	3.8±0.8
Lhunze	28.42	92.47	Steppe	3860	4.5±0.4	9.9±0.1
Pagri	27.73	89.08	Meadow	4302	1.8±0.4	7±0.7
Tuotuo-he	34.22	92.43	Steppe	4533	8.3±0.3	8.3±0.5
Zadoi	32.88	95.28	Meadow	4066	8.2±0.3	7.2±0.5
Qumalai	34.12	95.80	Meadow	4175	8.7±0.2	8.4±0.5
Yushu	33.00	96.97	Meadow	3681	4.5±0.7	5.7±0.6
Madoi	34.92	98.22	Steppe	4272	7.8±0.4	8.3±0.4
Qingshui-he	33.80	97.13	Meadow	4415	7.3±0.5	6.5±0.7
Shiqu	32.98	98.10	Meadow	4200	6.5±0.4	5.4±0.5
Maqin	34.48	100.23	Meadow	3719	8.2±0.3	9±0.3
Dari	33.75	99.65	Meadow	3968	6.9±0.4	6.8±0.4
Henan	34.73	101.60	Meadow	3500	8.9±0.4	8.6±0.7
Jigzhi	33.43	101.48	Meadow	3629	7±0.5	8±0.5

Maqu	34.00	102.08	Meadow	3471	5.6±0.4	8.7±0.3
Zoige	33.58	102.97	Meadow	3440	7.3±0.5	8.8±0.3
Hezuo	35.00	102.90	Meadow	2910	8.6±0.3	8.2±0.4
Sog	31.88	93.78	Shrub	4023	7.6±0.3	7.7±0.6
Dênqên	31.42	95.60	Meadow	3873	7.2±0.5	8.6±0.3
Nangqên	32.20	96.47	Shrub	3644	8.4±0.3	9.8±0.1
Qamdo	31.15	97.17	Shrub	3306	7.8±0.6	9.6±0.1
Dêgê	31.80	98.58	Meadow	3184	4.9±0.5	8.7±0.4
Garzê	31.62	100.00	Shrub	3394	3.8±0.7	7.9±0.6
Baima	32.93	100.75	Shrub	3530	7.6±0.3	9.1±0.2
Sêrtar	32.28	100.33	Meadow	3894	6.8±0.6	8.3±0.5
Dawu	30.98	101.12	Shrub	2957	6.5±0.5	9.7±0.1
Hongyuan	32.80	102.55	Meadow	3492	7.5±0.4	8.3±0.4
Lhari	30.67	93.28	Meadow	4489	0.5±0.2	2.3±0.7
Lhorong	30.75	95.83	Shrub	3640	3.5±0.6	8.5±0.4
Batang	30.00	99.10	Shrub	2589	4.2±0.7	9.3±0.2
Xinlong	30.93	100.32	Shrub	3000	6.2±0.6	9.5±0.2

Litang	30.00	100.27	Meadow	3949	6.8±0.5	10±0
Zogang	29.67	97.83	Shrub	3780	2.8±0.4	8.5±0.5
Daocheng	29.05	100.30	Meadow	3728	5.6±0.5	9.8±0.1
Shangri-la	27.83	99.70	Shrub	3276	1.6±0.4	8±0.3

Table S3. Partial correlation coefficients (R-value) of multiple regressions for relationship of SVG to rainy season onset (RSO), pre-SVG temperature (T_{-30d}) and precipitation (P_{-30d}) in each of the 67 stations during 2001-2013. $^{\#}P < 0.10$, $^{*}P < 0.05$, $^{**}P < 0.01$, $^{***}P < 0.001$; ns, not significant.

Weather stations	Latitude (°)	Longitude (°)	Vegetation types	Altitude (m)	RSO's R-value	T_{-30d}'s R-value	P_{-30d}'s R-value
Tuole	38.82	98.42	Meadow	3367	$0.50^{\#}$	-0.26^{ns}	-0.22^{ns}
Yeniugou	38.42	99.58	Meadow	3320	0.59^{*}	0.01^{ns}	-0.28^{ns}
Qilian	38.18	100.25	Meadow	2787	0.64^{*}	0.06^{ns}	-0.08^{ns}
Xiaozaohuo	36.80	93.68	Meadow	2767	0.22^{ns}	-0.30^{ns}	-0.41^{ns}
Dachaidan	37.85	95.35	Meadow	3173	$0.52^{\#}$	-0.32^{ns}	0.11^{ns}
Gangcha	37.33	100.13	Meadow	3302	0.47^{ns}	-0.12^{ns}	-0.20^{ns}
Menyuan	37.38	101.62	Meadow	2850	0.28^{ns}	-0.33^{ns}	$-0.52^{\#}$
Wushaoling	37.20	102.87	Meadow	3045	0.29^{ns}	-0.11^{ns}	-0.29^{ns}
Golmud	36.42	94.92	Meadow	2808	0.07^{ns}	-0.38^{ns}	-0.05^{ns}
Nomhon	36.43	96.43	Meadow	2790	-0.13^{ns}	-0.26^{ns}	-0.29^{ns}
Gonghe	36.27	100.62	Steppe	2835	0.33^{ns}	-0.68^{*}	-0.38^{ns}
Xining	36.73	101.75	Steppe	2295	0.57^{*}	-0.22^{ns}	$-0.49^{\#}$

Guide	36.03	101.43	Steppe	2237	$0.48^{\#}$	-0.31^{ns}	-0.26^{ns}
Wudaoliang	35.22	93.08	Steppe	4612	0.10^{ns}	$-0.54^{\#}$	-0.28^{ns}
Xinghai	35.58	99.98	Steppe	3323	0.73^{**}	-0.65^{*}	-0.02^{ns}
Guinan	35.58	100.73	Steppe	3120	$0.49^{\#}$	-0.38^{ns}	-0.04^{ns}
Tongren	35.52	102.02	Meadow	2491	0.63^{*}	-0.44^{ns}	0.00^{ns}
Gerze	32.15	84.42	Steppe	4415	0.59^{*}	0.16^{ns}	-0.25^{ns}
Baingoin	31.38	90.02	Steppe	4700	0.59^{*}	0.18^{ns}	0.04^{ns}
Amdo	32.35	91.10	Steppe	4800	0.70^{**}	-0.38^{ns}	-0.06^{ns}
Nagqu	31.48	92.07	Meadow	4507	0.44^{ns}	0.34^{ns}	-0.11^{ns}
Purang	30.28	81.25	Steppe	3900	-0.04^{ns}	-0.18^{ns}	-0.41^{ns}
Shenza	30.95	88.63	Steppe	4672	0.56^{*}	-0.37^{ns}	-0.06^{ns}
Damxung	30.48	91.10	Meadow	4200	0.69^{**}	0.37^{ns}	-0.30^{ns}
Lhatse	29.08	87.60	Steppe	4000	0.77^{**}	0.04^{ns}	-0.35^{ns}
Shigatse	29.25	88.88	Shrub	3836	0.89^{***}	-0.36^{ns}	0.32^{ns}
Nyêmo	29.43	90.17	Shrub	3809	0.74^{**}	-0.45^{ns}	$-0.49^{\#}$
Lhasa	29.67	91.13	Shrub	3649	0.03^{ns}	-0.44^{ns}	-0.77^{**}
Zetang	29.25	91.77	Shrub	3552	0.73^{**}	0.32^{ns}	-0.11^{ns}

Tingri	28.63	87.08	Steppe	4300	0.70^{**}	-0.21^{ns}	-0.45^{ns}
Gyantse	28.92	89.60	Steppe	4040	0.79^{**}	0.59^{*}	-0.25^{ns}
Nagarze	28.97	90.40	Steppe	4432	0.75^{**}	0.38^{ns}	-0.36^{ns}
Cona	27.98	91.95	Shrub	4280	0.19^{ns}	-0.3^{ns}	-0.29^{ns}
Lhunze	28.42	92.47	Steppe	3860	0.37^{ns}	$-0.49^{\#}$	-0.33^{ns}
Pagri	27.73	89.08	Meadow	4302	0.35^{ns}	$-0.48^{\#}$	0.15^{ns}
Tuotuo-he	34.22	92.43	Steppe	4533	0.46^{ns}	-0.61^{*}	-0.38^{ns}
Zadoi	32.88	95.28	Meadow	4066	0.70^{**}	$-0.54^{\#}$	-0.24^{ns}
Qumalai	34.12	95.80	Meadow	4175	0.72^{**}	-0.41^{ns}	-0.02^{ns}
Yushu	33.00	96.97	Meadow	3681	0.87^{***}	-0.31^{ns}	$0.52^{\#}$
Madoi	34.92	98.22	Steppe	4272	0.31^{ns}	-0.12^{ns}	-0.23^{ns}
Qingshui-he	33.80	97.13	Meadow	4415	-0.09^{ns}	-0.63^{*}	0.44^{ns}
Shiqu	32.98	98.10	Meadow	4200	0.22^{ns}	-0.46^{ns}	-0.09^{ns}
Maqin	34.48	100.23	Meadow	3719	0.20^{ns}	$-0.54^{\#}$	-0.62^{*}
Dari	33.75	99.65	Meadow	3968	0.65^{*}	-0.13^{ns}	0.22^{ns}
Henan	34.73	101.60	Meadow	3500	0.12^{ns}	0.04^{ns}	-0.07^{ns}
Jigzhi	33.43	101.48	Meadow	3629	0.37^{ns}	-0.12^{ns}	-0.28^{ns}

Maqu	34.00	102.08	Meadow	3471	0.56^{*}	-0.02^{ns}	-0.01^{ns}
Zoige	33.58	102.97	Meadow	3440	0.77^{**}	-0.18^{ns}	0.10^{ns}
Hezuo	35.00	102.90	Meadow	2910	0.05^{ns}	-0.39^{ns}	0.19^{ns}
Sog	31.88	93.78	Shrub	4023	-0.02^{ns}	0.02^{ns}	-0.42^{ns}
Dênqên	31.42	95.60	Meadow	3873	-0.37^{ns}	0.05^{ns}	-0.37^{ns}
Nangqên	32.20	96.47	Shrub	3644	0.69^{**}	$-0.53^{\#}$	-0.38^{ns}
Qamdo	31.15	97.17	Shrub	3306	-0.68^{*}	0.59^{*}	-0.64^{*}
Dêgê	31.80	98.58	Meadow	3184	0.45^{ns}	-0.09^{ns}	-0.22^{ns}
Garzê	31.62	100.00	Shrub	3394	0.39^{ns}	-0.16^{ns}	-0.43^{ns}
Baima	32.93	100.75	Shrub	3530	0.63^{*}	0.12^{ns}	-0.34^{ns}
Sêrtar	32.28	100.33	Meadow	3894	0.21^{ns}	-0.20^{ns}	-0.24^{ns}
Dawu	30.98	101.12	Shrub	2957	-0.04^{ns}	-0.42^{ns}	-0.45^{ns}
Hongyuan	32.80	102.55	Meadow	3492	0.06^{ns}	-0.70^{**}	-0.58^{*}
Lhari	30.67	93.28	Meadow	4489	-0.29^{ns}	0.12^{ns}	0.69^{**}
Lhorong	30.75	95.83	Shrub	3640	$0.50^{\#}$	0.07^{ns}	-0.16^{ns}
Batang	30.00	99.10	Shrub	2589	0.46^{ns}	0.32^{ns}	0.35^{ns}
Xinlong	30.93	100.32	Shrub	3000	$0.52^{\#}$	0.13^{ns}	0.01^{ns}

Litang	30.00	100.27	Meadow	3949	0.67*	0.68*	0.31ns
Zogang	29.67	97.83	Shrub	3780	0.24ns	0.36ns	-0.24ns
Daocheng	29.05	100.30	Meadow	3728	0.70**	0.53#	-0.17ns
Shangri-la	27.83	99.70	Shrub	3276	0.73**	0.08ns	0.05ns

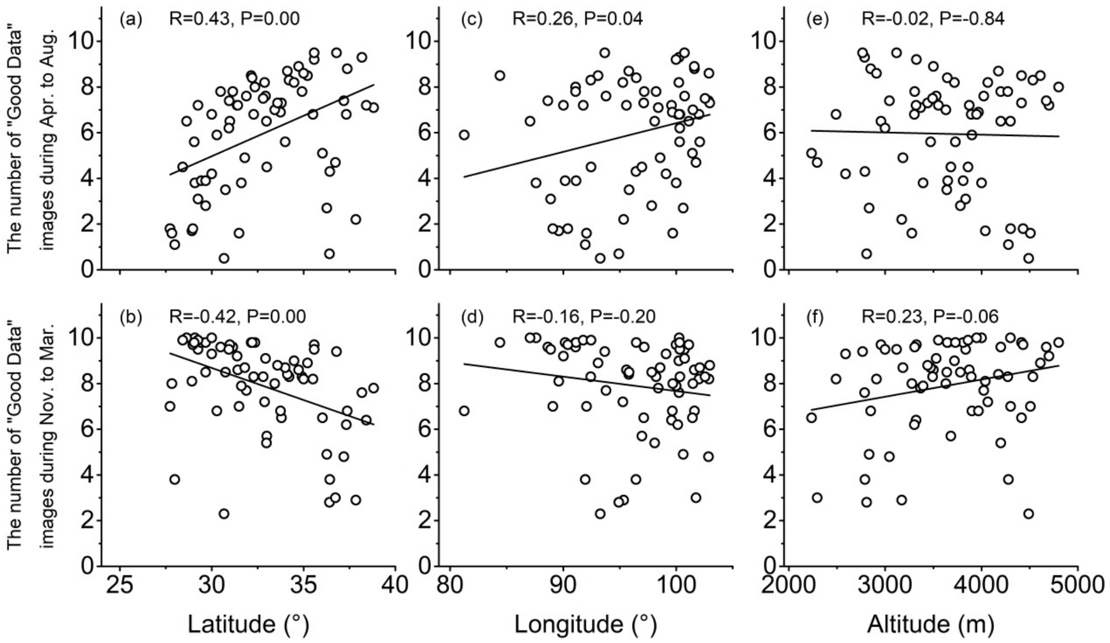

Fig. S1. Variations in the average number of 16-day ‘Good Data’ images in the growing season (a,c,e) and non-growing season (b,d,f) along gradients of latitude, longitude and altitude. The data were from Table S2.

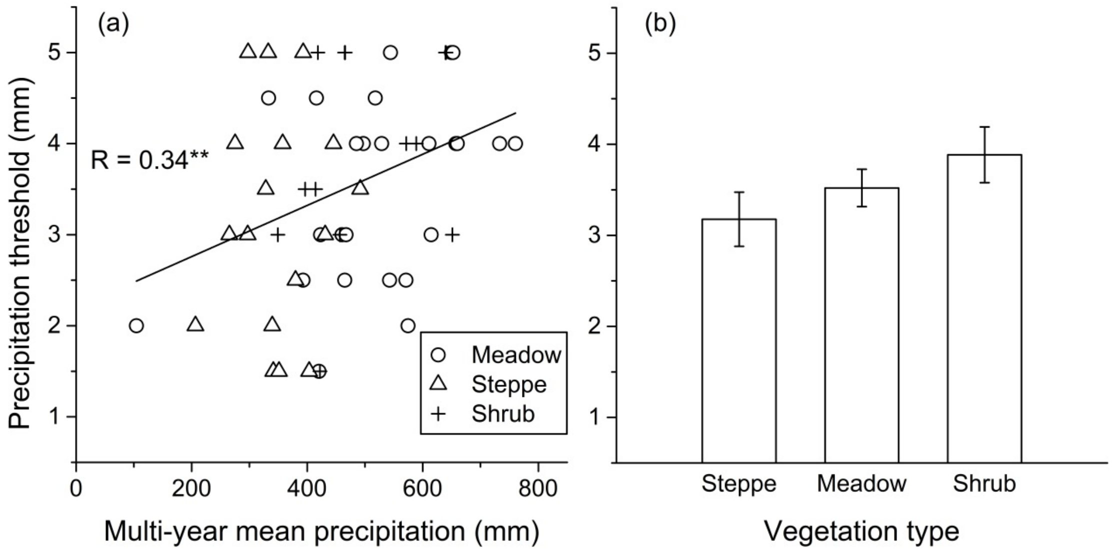

Fig. S2 The estimated optimum precipitation thresholds tended to increase with increasing annual precipitation (a, $^{**}P < 0.01$), with mean values ranging from 3.2-3.5 mm in steppes and meadows to 4.0 mm in shrubs (b). (b) Bar lines indicate standard error of the mean.

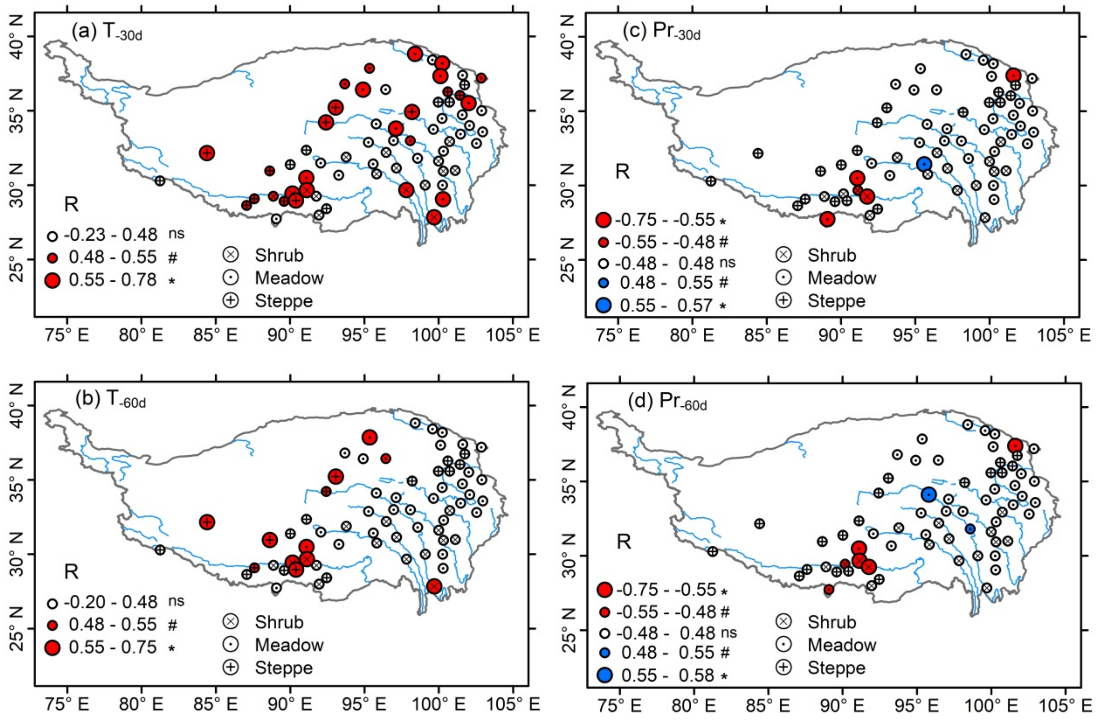

Fig. S3 Interannual change trends in pre-SVG temperatures (a, T_{-30d}; b, T_{-60d}) and precipitation (c, P_{-30d}; d, P_{-60d}) during 2001-2013. Different sizes of the circles indicate the significant levels of correlation coefficients (R-values) (*, $P < 0.05$; #, $P < 0.10$; ns, not significant). Red circles, warming/drying. Blue circles, cooling/wetting. The map was drawn with the vector data available free online at http://www.geodata.cn/, using ArcGIS 10.3 for Desktop.

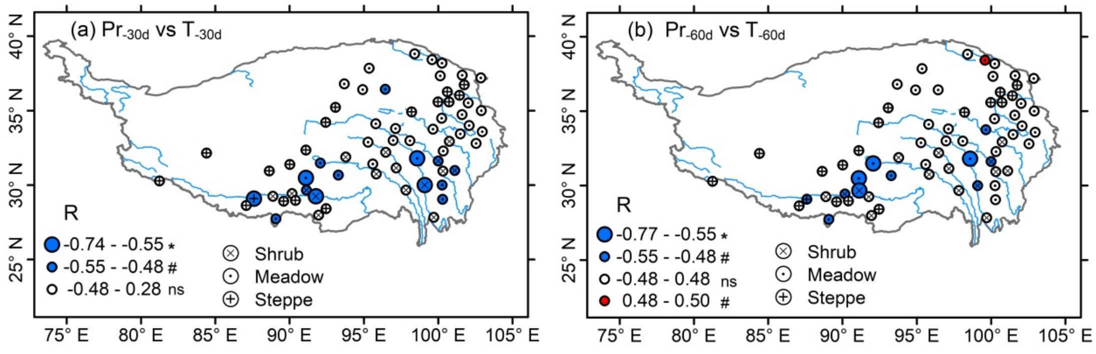

Fig. S4 Correlation coefficients (R-value) between pre-SVG precipitation (P_{-30d}, P_{-60d}) and mean temperature (T_{-30d}, T_{-60d}) for each of the 67 sites during 2001-2013. Different sizes of the circles indicate the significant levels of correlation coefficients (*, $P < 0.05$; #, $P < 0.10$; ns, not significant). The map was drawn with the vector data available free online at http://www.geodata.cn/, using ArcGIS 10.3 for Desktop.
